# Bullous Skin Manifestations of *Mycoplasma pneumoniae* Infection: A Case Series

**DOI:** 10.1177/2324709617727759

**Published:** 2017-09-08

**Authors:** Senthil Velan Bhoopalan, Vonita Chawla, Mary Beth Hogan, Nevin W. Wilson, Samrat U. Das

**Affiliations:** 1 University of Nevada, Las Vegas Campus, Las Vegas, NV, USA

**Keywords:** *Mycoplasma pneumoniae*, Stevens-Johnson syndrome, bullous, blisters, corticosteroids, IVIG

## Abstract

Bullous skin lesions are uncommon in children. While it is well known that *Mycoplasma* infections are associated with papular skin manifestations, bullous skin lesions are not commonly reported. *Mycoplasma pneumoniae* is a very common bacterial pathogen causing respiratory tract infection in children and adults. We report 2 children with serology-confirmed *Mycoplasma* infection who were hospitalized for blistering skin lesions. Both of our patients responded well to corticosteroids and one of them required intravenous immunoglobulin. The aim of this case report is to raise awareness that *Mycoplasma pneumoniae* infection can present with bullous skin lesions, and to briefly review the pathophysiology, diagnosis, and management of the skin manifestation of *Mycoplasma* infection.

## Introduction

Bullous skin lesions are uncommon in children. They are a heterogeneous group of disorders characterized by blisters in skin, with or without involvement of mucous membrane. Skin blisters can be caused by various reasons such as infection, genetic conditions, and autoimmune reactions.^1,2^ *Mycoplasma pneumoniae* is a bacterial pathogen that typically causes community-acquired pneumonia associated with nonblistering maculopapular skin lesions.^3,4^ However, in this case series, we report 2 cases of bullous skin lesions associated with *Mycoplasma* infections. The case report aims to raise awareness of *M pneumoniae* infection as a cause for acute-onset blistering skin lesions in pediatric patients. *M. pneumoniae* is an obligate human pathogen that causes community-acquired pneumonia, especially in children older than 5 years of age. About 20% to 50% of cases of community-acquired pneumonia among teenagers and military recruits are estimated to be caused by *M. pneumoniae.*^3,4^ Although majority of the lung infections caused by *M. pneumoniae* are mild and self-limited, they can be associated with a wide variety of extrapulmonary manifestations.^5^ While pneumonia and other respiratory presentations are caused by direct infection, the extrapulmonary symptoms are mediated by one of the following three mechanisms: direct infection (eg, pericarditis, arthritis, aseptic meningitis, encephalitis, and myelitis), immune-mediated damage caused by cross-reacting antibodies or immune complexes (eg, hemolytic anemia, conjunctivitis, iritis, uveitis, myocarditis), and vascular occlusion by either direct infection with bacteria or by vasculitis (aortic thrombus, pancreatitis, splenic infarct, pulmonary embolism, priapism, renal artery embolism, thalamic necrosis).^5^ Mucocutaneous manifestations of *Mycoplasma* infection can present as erythema nodosum, cutaneous leukocytoclastic vasculitis, erythema multiforme, Stevens-Johnson syndrome (SJS), toxic epidermal necrolysis (TEN), Fuchs syndrome, and very rarely subcorneal pustular dermatosis.^6^

## Case 1

An 8-year-old female with no significant past medical history presented with diffuse erythematous nonpruritic rash anteriorly over both thighs and laterally over the right thigh, which started as a red bump on the night prior to admission. The rash worsened over the course of a few hours progressing to blisters on top of the rash. She reported a few episodes of coughing and runny nose 2 to 3 weeks prior to admission. However, she was not treated with any antibiotics, and at the time of admission she did not have signs or symptoms of respiratory tract infection. She came back from a 10-day trip to California with her family and cousins but denies having any sick contacts. While she reported 1 day of loose stool, she did not take any antibiotics or any other medications in the last few weeks. No dysuria or eye pain was reported.

Physical examination revealed normal vital signs, and a tender erythematous rash with diffuse borders, approximated 10 cm by 15 cm on the anterolateral aspect of right thigh and another similar appearing lesion about 5 cm by 5 cm on the anterior aspect of left thigh. Tense blisters were noted over these erythematous areas. Her oral mucosa did not have any lesions. Her lung and cardiovascular examination was also unremarkable. While in the hospital her rash continued to increase in size and blisters worsened with increase in size and number. Initial laboratory examination showed a normal complete blood count and comprehensive metabolic panel.

Since the rash was worsening and associated with blistering, we sent a skin biopsy that showed normal immunofluorescence studies, ruling out cutaneous autoimmune conditions such as bullous pemphigoid. Histological examination revealed focal epidermal necrosis with perivascular lymphocytic infiltrate suggestive of subacute erythema multiforme-like reaction. Results of blood test for hepatitis A, B, and C viruses, Epstein-Barr virus (EBV), human herpes virus 6 (HHV6), herpes simplex virus (HSV), and cytomegalovirus (CMV) were negative. Her *Mycoplasma pneumoniae*–specific IgM titer came back elevated at 1823 U/mL (positive > 950 U/mL). She was diagnosed with *Mycoplasma*-associated bullous erythema multiforme. She responded well to corticosteroids and did not have any mucosal involvement during the entire course of her illness. No antibiotics were given as the patient did not have any respiratory symptoms. She was subsequently discharged after the skin lesions showed improvement.

## Case 2

Our second patient is a 14-year-old male with no significant past medical history who presented with 2 days of painful sores in his mouth. While he did not have any illness during the preceding few weeks, he had a fever on the day of admission. He denies any recent medications, sick contacts, or travel.

At the time of admission he had a temperature of 39°C, heart rate of 112 beats/minute, and blood pressure of 108/82 mm Hg. He had numerous papular rashes on an erythematous base each ranging from 0.5 cm to 1 cm in diameter, on the dorsal aspect of both forearms and the anterior aspect of right thigh. His respiratory and cardiovascular system did not show any signs of abnormality. Multiple painful oral lesions associated with diffuse oral mucosal erythema and edema were noted. He was diagnosed with presumed SJS and immediately started on intravenous methylprednisolone. The following day he developed pain in his right eye while the rash continued to spread with some coalescence of borders. In light of the worsening lesions, intravenous immunoglobulin (IVIG) therapy was initiated, along with an ophthalmology consult for concerns about ocular manifestations of SJS. Eye exam was normal, except for conjunctival injection. Our patient also developed genital mucosal and skin lesions as well. While HSV polymerase chain reaction (PCR) and influenza rapid antigen test came back negative, he showed *M. pneumoniae* IgM titer of 1217 U/mL (positive > 950). He did not require any antibiotics as there were no respiratory complaints. Skin biopsy was deferred as the patient continued to make progress on IVIG. Similar to the previous case report, he tested negative for hepatitis A, B, and C viruses, EBV, HHV6, HSV, and CMV. After a 2-week course involving treatment with both steroids and IVIG, he was discharged as his rash and mucositis had significantly improved.

## Discussion

The two patients with presumed *Mycoplasma* infection highlight the protean manifestations of *M pneumoniae* infection. A major limitation in this case report is the absence of convalescent titers, cold titers, and PCR testing. While IgM testing by itself has a specificity of only 92% compared to IgM combined with *Mycoplasma* PCR, which has a specificity of 100% and sensitivity of 98%.^7^ However, both our patients tested negative for other infectious causes of bullous skin lesions such as HSV, CMV, EBV, and hepatitis viruses while only the *Mycoplasma* IgM was elevated. The positive IgM titers (in the absence of convalescent titers) suggest either acute or very recent infection.^7^ IgM testing, although highly sensitive (81%), can remain positive for a few weeks after an acute infection.^7,8^

Bullous skin lesions are not common in children. The differential diagnosis for bullous skin lesions include infectious causes such as bullous impetigo, bullous tinea, eczema herpeticum, bullous scabies, chickenpox, and herpes simplex infection; infection- and drug-associated causes such as SJS and TEN; hereditary conditions such as epidermolysis bullosa, Kindler syndrome, and incontinenti pigmenti; and autoimmune conditions such as linear IgA bullous dermatitis, bullous pemphigoid, and pemphigoid vulgaris.^1,2^ The cases discussed above highlight that *Mycoplasma* infections can also present with blistering skin lesions in children. While *M. pneumoniae* infections have been reported to result in extrapulmonary manifestations in 25% to 33% of the cases, reports of bullous skin lesions are rare.^5,9^ While skin and mucosal manifestations are one of the most common extrapulmonary features of *M. pneumonia*, majority of the lesions are exanthematous nonbullous in nature.^9^ Less frequently erythema nodosum, erythema multiforme, SJS, TEN, Fuch’s syndrome, and cutaneous leukocytoclastic vasculitis are noted.^5,10,11^ Onset of these extrapulmonary manifestations are reported to be quite variable in relation to pulmonary signs and symptoms. Occasionally, they are reported even in the absence of any respiratory symptoms. The aforementioned case reports highlight that *Mycoplasma* infections need to be considered even in children with bullous lesions as initial presentation.

The mechanisms behind these skin and mucosal lesions have not been completely understood, although evidence predominantly suggests an autoimmune response.^9,12^ Some authors also suggest vascular injury caused by immune complex deposition.^5^ Superficial dermal perivasculitis is noted during the initial phase of SJS suggesting vascular injury might play an important role. However, *M. pneumoniae* has also been isolated from skin blisters by multiple independent investigators raising the possibility of cutaneous infection.^13-15^ At the cellular level, Fas ligand (FasL)–mediated epidermal apoptosis and cytotoxic T-cell-mediated cell death have both been reported in SJS and TEN.^16^ The wide range of skin presentation and the multiple mechanisms reported suggest a complex interaction between host- and pathogen-dependent factors.

Patients typically present 1 to 3 weeks after exposure, although some patients may not report any history of respiratory issues. As noted in our case report, skin lesions are usually associated with a prodrome of general malaise, fatigue, and fever. Oral, pharyngeal, respiratory, ocular, genitourinary, and rectal mucosa have all been reported to be involved.^17^ Clinical presentation depending on the affected mucosa can result in oral pain, dysphagia, dyspnea, photophobia, ocular pain, dysuria, dyschezia, hematochezia, and rectal pain. Skin lesions in majority of the patients present as an urticarial lesion but other presentations such as target lesions, macular rash, blisters, and bullae are much rarer. As reported here, presentation can also involve mucosal membrane, along with skin lesions. While traditionally erythema multiforme, SJS, and TEN were considered to be diseases along the same spectrum with different severity, current understanding suggests erythema multiforme (with or without bullous) is a separate entity with lower morbidity compared to SJS/TEN.^18^

Differential diagnosis to consider are viral infections with HSV, EBV, CMV, hepatitis B virus and bacterial infections such as *Streptococcus pyogenes* and *Staphylococcus aureus*, drug-mediated SJS/TEN, and Kawasaki disease. Systemic disease such as inflammatory bowel diseases can also present with a similar clinical picture. While history plays a major role in diagnosis, these patients are also typically treated with antibiotics prior to being seen in the emergency room making it tricky. Diagnosis can be confirmed by serology and/or PCR where available.^19,20^

Management of moderate skin and mucosal involvement is typically with intravenous glucocorticoids, with significant supportive care. However, in some patients steroids may not be adequate enough to stop the progression of the disease as evident in our second patient. Patients with severe skin and mucosal involvement are also given high-dose IVIG (1-2 g/kg) during the initial phase of disease.^21^ IVIG can be repeated up to 3 times as needed. While a few centers have reported using cyclosporine and plasmapheresis in SJS/TEN, their role has not yet been completely established. Similarly, the effect of treating *Mycoplasma* infection on the clinical course of SJS/TEN is not clearly understood. However, it would be prudent to treat any acute symptomatic infection. None of our patients were treated with azithromycin as they were not symptomatic with respiratory illness.

To conclude, this case series highlights that bullous skin lesions, with or without mucosal lesions, are important and occasionally underdiagnosed manifestations of *Mycoplasma* infections. Early diagnosis is important since these patients can have severe mucosal and systemic involvement, with potential life-threatening consequences, which might require IVIG as noted in one of our patients. In addition, prompt consultation with an ophthalmologist is important to manage ocular complications such as conjunctivitis, conjunctival ulceration, corneal scarring, epithelial sloughing, anterior uveitis, and corneal perforation. In addition, this series also highlights that patients can present without any preceding signs or symptoms of respiratory involvement suggesting that a high index of suspicion is needed to diagnose *Mycoplasma* infection. While our patients were not treated with antibiotics, they all responded well to either steroids alone or steroids and IVIG without any postinfectious sequelae.

## References

[1] Yeh SW Ahmed B Sami N Razzaque Ahmed A Blistering disorders: diagnosis and treatment. Dermatol Ther. 2003;16:214-223. 14510878 10.1046/j.1529-8019.2003.01631.x

[2] Tamesis ME Morel KD Bullous disorders of childhood. In: Hogeling M, ed. Case-Based Inpatient Pediatric Dermatology. Berlin, Germany: Springer; 2016:205-220.

[3] Foy HM Kenny GE Cooney MK Allan ID Long-term epidemiology of infections with Mycoplasma pneumoniae. J Infect Dis. 1979;139:681-687. 448195 10.1093/infdis/139.6.681

[4] Foy HM Grayston JT Kenny GE Alexander ER McMahan R Epidemiology of *Mycoplasma pneumoniae* infection in families. JAMA. 1966;197:859-866. 5952772

[5] Narita M Classification of extrapulmonary manifestations due to *Mycoplasma pneumoniae* infection on the basis of possible pathogenesis. Front Microbiol. 2016;7:23. 26858701 10.3389/fmicb.2016.00023 PMC4729911

[6] Bohelay G Duong TA Ortonne N Chosidow O Valeyrie-Allanore L Subcorneal pustular dermatosis triggered by *Mycoplasma pneumoniae* infection: a rare clinical association. J Eur Acad Dermatol Venereol. 2015;29:1022-1025. 24650287 10.1111/jdv.12446

[7] Waris ME Toikka P Saarinen T et al Diagnosis of *Mycoplasma pneumoniae* pneumonia in children. J Clin Microbiol. 1998;36:3155-3159. 9774556 10.1128/jcm.36.11.3155-3159.1998 PMC105292

[8] Thurman KA Walter ND Schwartz SB et al Comparison of laboratory diagnostic procedures for detection of *Mycoplasma pneumoniae* in community outbreaks. Clin Infect Dis. 2009;48:1244-1249. 19331586 10.1086/597775

[9] Narita M Pathogenesis of extrapulmonary manifestations of *Mycoplasma pneumoniae* infection with special reference to pneumonia. J Infect Chemother. 2010;16:162-169. 20186455 10.1007/s10156-010-0044-x

[10] Copps SC Allen VD Sueltmann S Evans AS A community outbreak of Mycoplasma pneumonia. JAMA. 1968;204:123-128. 5694534

[11] Canavan TN Mathes EF Frieden I Shinkai K *Mycoplasma pneumoniae*–induced rash and mucositis as a syndrome distinct from Stevens-Johnson syndrome and erythema multiforme: a systematic review. J Am Acad Dermatol. 2015;72:239-245. 25592340 10.1016/j.jaad.2014.06.026

[12] Letko E Papaliodis DN Papaliodis GN Daoud YJ Ahmed AR Foster CS Stevens-Johnson syndrome and toxic epidermal necrolysis: a review of the literature. Ann Allergy Asthma Immunol. 2005;94:419-436. 15875523 10.1016/S1081-1206(10)61112-X

[13] Stutman HR Stevens-Johnson syndrome and *Mycoplasma pneumoniae*: evidence for cutaneous infection. J Pediatr. 1987;111:845-847. 3119808 10.1016/s0022-3476(87)80200-7

[14] Meseguer MA de Rafael L Vidal ML Stevens-Johnson syndrome with isolation of *Mycoplasma pneumoniae* from skin lesions. Eur J Clin Microbiol Infect Dis. 1986;5:167-168. 10.1007/BF02013977 3087748

[15] Lyell A Gordon AM Dick HM Sommerville RG Mycoplasmas and erythema multiforme. Lancet. 1967;290:1116-1118. 10.1016/s0140-6736(67)90620-4 4168557

[16] Khalili B Bahna SL Pathogenesis and recent therapeutic trends in Stevens-Johnson syndrome and toxic epidermal necrolysis. Ann Allergy Asthma Immunol. 2006;97:272-281. 17042130 10.1016/S1081-1206(10)60789-2

[17] Wetter DA Camilleri MJ Clinical, etiologic, and histopathologic features of Stevens-Johnson syndrome during an 8-year period at Mayo Clinic. Mayo Clin Proc. 2010;85:131-138. 20118388 10.4065/mcp.2009.0379 PMC2813820

[18] Schalock PC Dinulos JG Pace N Schwarzenberger K Wenger JK Erythema multiforme due to *Mycoplasma pneumoniae* infection in two children. Pediatr Dermatol. 2006;23:546-555. 17155996 10.1111/j.1525-1470.2006.00307.x

[19] Zhang L Zong ZY Liu YB Ye H Lv XJ PCR versus serology for diagnosing *Mycoplasma pneumoniae* infection: a systematic review & meta-analysis. Indian J Med Res. 2011;134:270-280. 21985809 PMC3193707

[20] Morozumi M Hasegawa K Chiba N et al Application of PCR for *Mycoplasma pneumoniae* detection in children with community-acquired pneumonia. J Infect Chemother. 2004;10:274-279. 16163461 10.1007/s10156-004-0338-y

[21] Momin SB Review of intravenous immunoglobulin in the treatment of Stevens-Johnson syndrome and toxic epidermal necrolysis. J Clin Aesthet Dermatol. 2009;2(2):51-58. PMC2958184 20967183

